# Supplementary figures and images for: Asymmetrical Inheritance of Plasmids Depends on Dynamic Cellular Geometry and Volume Exclusion Effects
Source: PLoS One. 2015 Oct 15;10(10):e0139443. doi: 10.1371/journal.pone.0139443 (PMC4607505; doi:10.1371/journal.pone.0139443)

**A)**

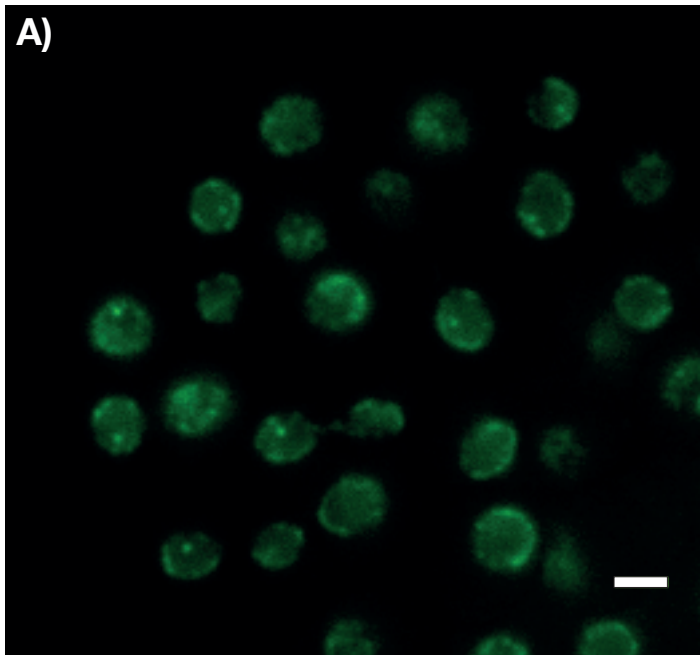

**B)**

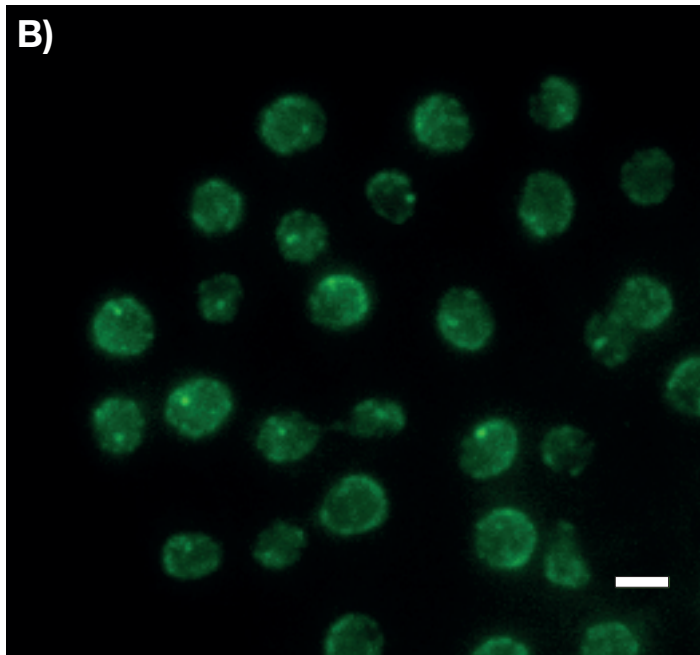

Supplement: S1 Fig — Example of single time points of GA1320 cells. Given both the nuclear pore and plasmid are marked with GFP, direct plasmid diffusion measurements can become difficult. A) A single Z-stack taken from a stack of 6 separated by 0.5 μm. B) Maximum intensity projection of all 6 Z-Stack slices. (PDF) [file pone.0139443.s001.pdf]

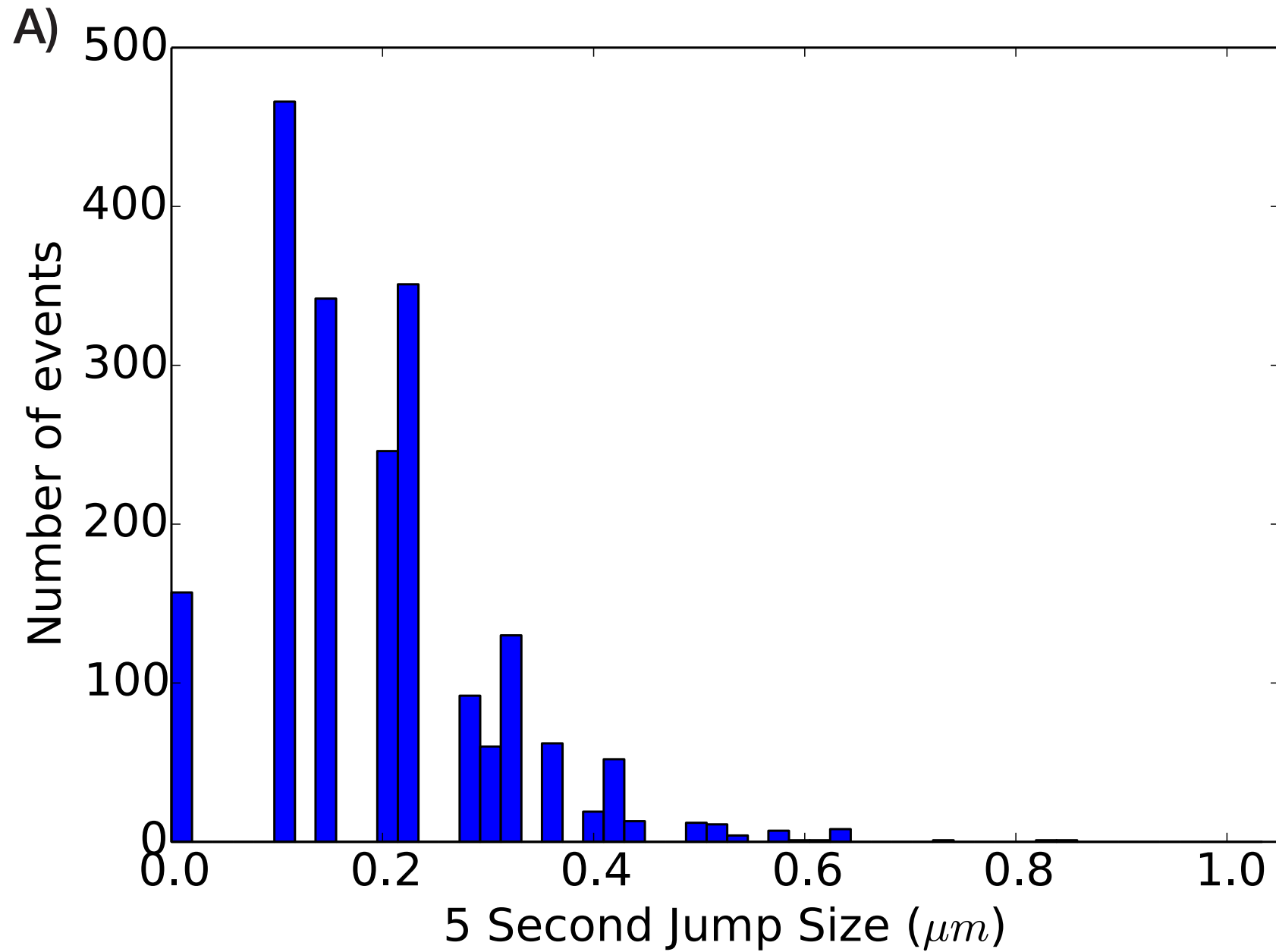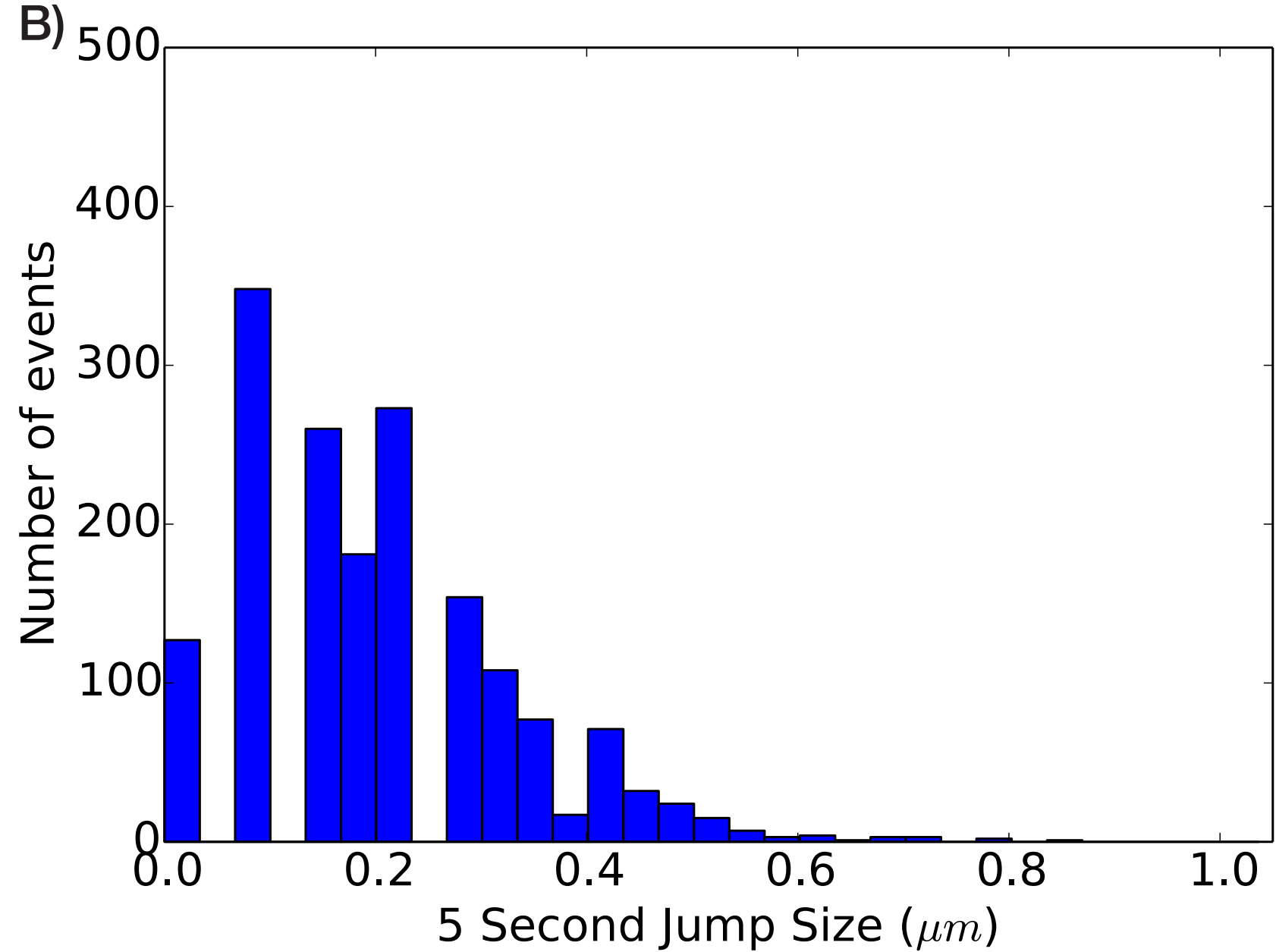

Supplement: S2 Fig — Histograms showing the frequency of jump sizes of plasmids sampled at 5 second increments from 24 distinct cells in interphase (subplot a) and anaphase (subplot b). Histogram bin sizes were calculated according to the Freedman-Diaconis method [27]. Gaps in the histogram occur due to the plasmid jumping over discrete pixels. Owing to the difficulty in accurately estimating diffusion coefficients from this data, our study investigated a range of diffusion rates in simulations. (PDF) [file pone.0139443.s002.pdf]
