# Supplementary material for: Asymmetrical Inheritance of Plasmids Depends on Dynamic Cellular Geometry and Volume Exclusion Effects
Source: PLoS One. 2015 Oct 15;10(10):e0139443. doi: 10.1371/journal.pone.0139443 (PMC4607505; doi:10.1371/journal.pone.0139443)
Supplement: S1 SpreadSheet — A spread sheet containing the exact measurements for each of the geometries used in the simulations. (PDF) [file pone.0139443.s005.pdf]

S1 Spread sheet - Denton J. A., Ghosh, A. and Marquez-Lago, T.T.

* Notes:

1. All length measurements are in microns, and time measurements in seconds

2. In cases where the bridge length is not seen in the microscopy, a small bridge of 0.05 microns is recorded. A value of 0 causes errors when assembling the geometries.

3. In cases where the daughter is not visible in microscopy, a small daughter is appended to the mother. This ensures that the geometries can be reliably constructed.

4. Zero sized daughters can sometimes give rise to some imaginary numbers in trigonometric functions used in the geometry constructions.

5. In such scenarios, the daughter length is chosen asv 0.15 microns, and its width is chosen as the first measured width from microscopy.

6. Geometry measurements sometimes continue past the point of karyofission. We have demarcated the point where karyofission occurs in each spreadsheet.

Cell 1

Time(s)	Mother Length	Daughter Length	Bridge Length	Mother Width	Daughter Width	Bridge Width	
0.00E+00	2.58E+00	1.50E-01	5.00E-02	2.38E+00	5.15E-01	5.15E-01	
3.00E+01	2.83E+00	1.50E-01	5.00E-02	2.38E+00	5.15E-01	5.15E-01	
6.00E+01	2.89E+00	1.50E-01	5.00E-02	2.22E+00	5.15E-01	5.15E-01	
9.00E+01	3.30E+00	1.50E-01	5.00E-02	2.22E+00	5.15E-01	5.15E-01	
1.20E+02	3.52E+00	1.50E-01	5.00E-02	2.19E+00	5.15E-01	5.15E-01	
1.50E+02	3.22E+00	6.60E-01	5.00E-02	2.19E+00	5.15E-01	5.15E-01	
1.80E+02	3.51E+00	5.28E-01	5.00E-02	2.17E+00	4.90E-01	4.90E-01	
2.10E+02	3.59E+00	6.79E-01	5.00E-02	2.19E+00	6.60E-01	6.60E-01	
2.40E+02	3.32E+00	7.38E-01	5.00E-02	2.01E+00	6.82E-01	6.82E-01	
2.70E+02	3.68E+00	8.47E-01	5.00E-02	2.00E+00	6.82E-01	6.82E-01	
3.00E+02	3.54E+00	1.05E+00	5.00E-02	2.02E+00	8.47E-01	8.47E-01	
3.30E+02	3.46E+00	1.17E+00	5.00E-02	2.03E+00	8.30E-01	6.70E-01	
3.60E+02	3.34E+00	1.17E+00	2.33E-01	2.00E+00	1.00E+00	1.00E+00	
3.90E+02	3.27E+00	1.21E+00	3.30E-01	1.89E+00	1.05E+00	8.30E-01	
4.20E+02	3.31E+00	1.16E+00	3.71E-01	2.05E+00	1.18E+00	1.01E+00	
4.50E+02	3.27E+00	1.54E+00	1.60E-01	2.05E+00	1.18E+00	1.01E+00	
4.80E+02	3.36E+00	1.37E+00	3.71E-01	2.00E+00	1.05E+00	6.60E-01	
5.10E+02	3.55E+00	1.34E+00	2.26E-01	2.19E+00	1.50E+00	8.45E-01	
5.40E+02	3.42E+00	1.34E+00	3.67E-01	2.05E+00	1.18E+00	6.60E-01	
5.70E+02	3.10E+00	1.73E+00	2.33E-01	2.05E+00	8.93E-01	6.82E-01	
6.00E+02	3.06E+00	2.22E+00	1.70E-01	2.22E+00	1.05E+00	8.47E-01	
6.30E+02	3.14E+00	1.73E+00	2.33E-01	2.05E+00	1.37E+00	8.30E-01	
6.60E+02	2.90E+00	1.68E+00	3.67E-01	1.85E+00	1.50E+00	8.45E-01	
6.90E+02	3.19E+00	1.73E+00	3.40E-01	2.09E+00	1.54E+00	9.69E-01	
7.20E+02	3.22E+00	1.57E+00	3.76E-01	1.85E+00	1.37E+00	6.82E-01	
7.50E+02	3.36E+00	1.53E+00	2.40E-01	1.85E+00	1.50E+00	6.60E-01	
7.80E+02	3.21E+00	1.42E+00	5.00E-01	1.86E+00	1.57E+00	8.47E-01	
8.10E+02	3.09E+00	2.00E+00	1.70E-01	1.85E+00	1.67E+00	8.47E-01	
8.40E+02	3.22E+00	1.83E+00	3.40E-01	1.85E+00	1.67E+00	5.28E-01	
8.70E+02	3.02E+00	2.21E+00	1.70E-01	2.00E+00	1.53E+00	8.30E-01	
9.00E+02	3.10E+00	1.90E+00	3.30E-01	1.86E+00	1.53E+00	8.47E-01	
9.30E+02	2.62E+00	1.69E+00	8.93E-01	1.86E+00	1.69E+00	6.60E-01	
9.60E+02	2.78E+00	2.10E+00	3.40E-01	2.19E+00	1.37E+00	5.00E-01	
9.90E+02	2.78E+00	1.85E+00	3.40E-01	1.85E+00	1.50E+00	6.82E-01	
1.02E+03	2.71E+00	2.22E+00	5.00E-01	2.02E+00	1.67E+00	3.40E-01	
1.05E+03	2.58E+00	2.19E+00	6.82E-01	2.02E+00	1.69E+00	3.80E-01	
1.08E+03	2.58E+00	2.17E+00	6.82E-01	2.00E+00	1.82E+00	3.40E-01	
1.11E+03	2.54E+00	2.10E+00	6.91E-01	1.85E+00	1.73E+00	1.73E+00	Karyofission
1.14E+03	2.58E+00	1.94E+00	1.12E+00	2.00E+00	1.95E+00	1.95E+00	
1.17E+03	2.41E+00	2.31E+00	1.12E+00	2.22E+00	1.57E+00	1.57E+00	
1.20E+03	2.58E+00	1.95E+00	1.21E+00	2.05E+00	1.73E+00	1.73E+00	

Cell 2

Time(s)	Mother Length	Daughter Length	Bridge Length	Mother Width	Daughter Width	Bridge Width	
0.00E+00	2.47E+00	1.50E-01	5.00E-02	2.09E+00	7.47E-01	7.47E-01	
3.00E+01	2.75E+00	1.50E-01	5.00E-02	2.23E+00	7.47E-01	7.47E-01	
6.00E+01	2.60E+00	1.50E-01	5.00E-02	2.16E+00	7.47E-01	7.47E-01	
9.00E+01	2.82E+00	1.50E-01	5.00E-02	2.08E+00	7.47E-01	7.47E-01	
1.20E+02	3.06E+00	1.50E-01	5.00E-02	1.94E+00	7.47E-01	7.47E-01	
1.50E+02	3.57E+00	1.50E-01	5.00E-02	1.79E+00	7.47E-01	7.47E-01	
1.80E+02	3.87E+00	1.50E-01	5.00E-02	2.08E+00	7.47E-01	7.47E-01	
2.10E+02	3.94E+00	1.50E-01	5.00E-02	1.79E+00	7.47E-01	7.47E-01	
2.40E+02	3.96E+00	1.50E-01	5.00E-02	2.01E+00	7.47E-01	7.47E-01	
2.70E+02	4.10E+00	1.50E-01	5.00E-02	2.16E+00	7.47E-01	7.47E-01	
3.00E+02	3.83E+00	5.25E-01	5.00E-02	1.94E+00	7.47E-01	7.47E-01	
3.30E+02	3.43E+00	6.79E-01	5.00E-02	2.00E+00	5.99E-01	5.99E-01	
3.60E+02	3.58E+00	8.36E-01	5.00E-02	2.03E+00	8.28E-01	8.28E-01	
3.90E+02	2.59E+00	1.17E+00	1.01E+00	2.00E+00	1.17E+00	3.76E-01	
4.20E+02	2.35E+00	1.73E+00	4.67E-01	2.03E+00	1.04E+00	5.19E-01	
4.50E+02	2.95E+00	1.30E+00	5.91E-01	2.10E+00	1.41E+00	3.71E-01	
4.80E+02	2.95E+00	1.53E+00	3.71E-01	1.89E+00	1.29E+00	1.07E+00	
5.10E+02	3.05E+00	1.66E+00	3.67E-01	2.11E+00	1.41E+00	5.99E-01	
5.40E+02	2.70E+00	1.79E+00	3.71E-01	1.89E+00	1.53E+00	5.99E-01	
5.70E+02	2.82E+00	1.79E+00	4.74E-01	1.80E+00	1.66E+00	4.67E-01	
6.00E+02	2.72E+00	1.99E+00	7.07E-01	2.13E+00	1.76E+00	3.76E-01	Karyofission
6.30E+02	2.70E+00	2.11E+00	7.51E-01	2.00E+00	1.64E+00	1.64E+00	
6.60E+02	2.49E+00	2.23E+00	9.69E-01	1.71E+00	1.53E+00	1.53E+00	
6.90E+02	2.47E+00	2.24E+00	1.29E+00	2.03E+00	1.66E+00	1.66E+00	
7.20E+02	2.36E+00	2.12E+00	1.71E+00	2.03E+00	1.65E+00	1.65E+00	

Cell 3

Time(s)	Mother Length	Daughter Length	Bridge Length	Mother Width	Daughter Width	Bridge Width	
0.00E+00	2.85E+00	1.50E-01	5.00E-02	2.17E+00	6.70E-01	6.70E-01	
3.00E+01	2.68E+00	1.50E-01	5.00E-02	2.34E+00	6.70E-01	6.70E-01	
6.00E+01	2.71E+00	1.50E-01	5.00E-02	2.22E+00	6.70E-01	6.70E-01	
9.00E+01	3.17E+00	1.50E-01	5.00E-02	2.19E+00	6.70E-01	6.70E-01	
1.20E+02	3.16E+00	1.50E-01	5.00E-02	2.17E+00	6.70E-01	6.70E-01	
1.50E+02	3.01E+00	8.47E-01	5.00E-02	1.99E+00	6.70E-01	6.70E-01	
1.80E+02	2.66E+00	6.91E-01	5.15E-01	2.00E+00	8.30E-01	5.25E-01	
2.10E+02	2.98E+00	5.19E-01	3.40E-01	2.17E+00	1.01E+00	8.30E-01	
2.40E+02	2.66E+00	1.17E+00	3.30E-01	2.17E+00	1.17E+00	8.30E-01	
2.70E+02	2.67E+00	1.33E+00	5.00E-01	2.19E+00	1.32E+00	6.60E-01	
3.00E+02	2.65E+00	1.17E+00	6.89E-01	2.00E+00	1.17E+00	6.91E-01	
3.30E+02	2.99E+00	1.34E+00	3.67E-01	2.17E+00	1.37E+00	8.47E-01	
3.60E+02	2.65E+00	1.85E+00	3.76E-01	2.17E+00	1.32E+00	1.01E+00	
3.90E+02	2.67E+00	1.67E+00	4.90E-01	1.84E+00	1.66E+00	8.30E-01	
4.20E+02	2.99E+00	2.00E+00	1.60E-01	2.01E+00	1.67E+00	8.93E-01	
4.50E+02	2.54E+00	2.02E+00	5.00E-01	1.84E+00	1.67E+00	6.79E-01	
4.80E+02	2.50E+00	2.38E+00	3.30E-01	1.67E+00	1.67E+00	4.67E-01	
5.10E+02	2.68E+00	2.38E+00	6.82E-01	1.84E+00	1.66E+00	1.60E-01	
5.40E+02	2.83E+00	2.05E+00	8.30E-01	1.99E+00	1.99E+00	3.30E-01	
5.70E+02	2.33E+00	2.34E+00	1.34E+00	1.86E+00	1.66E+00	3.40E-01	Karyofission
6.00E+02	2.50E+00	2.42E+00	1.33E+00	2.00E+00	1.82E+00	1.82E+00	
6.30E+02	2.50E+00	2.41E+00	1.54E+00	1.99E+00	2.00E+00	2.00E+00	
6.60E+02	2.49E+00	2.19E+00	1.67E+00	1.74E+00	1.69E+00	1.69E+00	

Cell 4

Time(s)	Mother Length	Daughter Length	Bridge Length	Mother Width	Daughter Width	Bridge Width	
0.00E+00	2.50E+00	1.50E-01	5.00E-02	1.86E+00	5.99E-01	5.99E-01	
3.00E+01	2.17E+00	1.50E-01	5.00E-02	2.01E+00	5.99E-01	5.99E-01	
6.00E+01	2.51E+00	1.50E-01	5.00E-02	2.02E+00	5.99E-01	5.99E-01	
9.00E+01	2.66E+00	1.50E-01	5.00E-02	2.00E+00	5.99E-01	5.99E-01	
1.20E+02	2.31E+00	7.38E-01	5.00E-02	2.17E+00	5.99E-01	5.99E-01	
1.50E+02	2.22E+00	3.80E-01	5.00E-02	2.16E+00	6.82E-01	6.82E-01	
1.80E+02	2.35E+00	5.28E-01	5.00E-02	2.00E+00	5.28E-01	5.28E-01	
2.10E+02	2.22E+00	7.38E-01	5.00E-02	2.22E+00	7.00E-01	7.00E-01	
2.40E+02	2.58E+00	5.28E-01	3.67E-01	2.19E+00	1.12E+00	7.38E-01	
2.70E+02	2.26E+00	1.07E+00	1.70E-01	2.21E+00	1.11E+00	9.69E-01	
3.00E+02	2.00E+00	9.69E-01	5.28E-01	2.10E+00	1.33E+00	8.93E-01	
3.30E+02	2.10E+00	1.12E+00	3.71E-01	2.11E+00	1.19E+00	7.38E-01	
3.60E+02	2.42E+00	1.26E+00	2.26E-01	2.05E+00	1.33E+00	8.45E-01	
3.90E+02	2.59E+00	1.05E+00	3.71E-01	1.86E+00	1.35E+00	5.28E-01	
4.20E+02	2.46E+00	1.41E+00	2.33E-01	2.01E+00	1.43E+00	8.30E-01	
4.50E+02	2.26E+00	1.26E+00	4.74E-01	1.86E+00	1.49E+00	5.25E-01	
4.80E+02	2.21E+00	1.64E+00	3.71E-01	1.88E+00	1.42E+00	8.45E-01	
5.10E+02	2.21E+00	1.71E+00	3.71E-01	1.73E+00	1.48E+00	6.99E-01	
5.40E+02	2.17E+00	1.58E+00	4.53E-01	1.85E+00	1.57E+00	1.10E+00	
5.70E+02	2.26E+00	1.85E+00	2.40E-01	1.85E+00	1.73E+00	6.82E-01	
6.00E+02	2.42E+00	1.42E+00	2.33E-01	1.86E+00	1.58E+00	5.99E-01	
6.30E+02	2.54E+00	1.73E+00	1.60E-01	1.69E+00	1.58E+00	7.38E-01	
6.60E+02	2.03E+00	1.73E+00	3.71E-01	1.82E+00	1.51E+00	7.38E-01	
6.90E+02	2.22E+00	1.89E+00	3.30E-01	1.85E+00	1.53E+00	4.74E-01	
7.20E+02	2.37E+00	1.63E+00	5.00E-01	1.83E+00	1.57E+00	5.15E-01	
7.50E+02	2.46E+00	1.89E+00	3.30E-01	1.69E+00	1.69E+00	5.00E-01	
7.80E+02	2.19E+00	1.90E+00	5.00E-01	1.67E+00	1.57E+00	5.00E-01	
8.10E+02	2.19E+00	2.07E+00	9.98E-01	1.83E+00	1.67E+00	3.67E-01	
8.40E+02	2.33E+00	1.84E+00	5.00E-01	1.66E+00	1.66E+00	3.67E-01	
8.70E+02	2.19E+00	2.00E+00	6.82E-01	1.69E+00	1.54E+00	1.70E-01	
9.00E+02	2.42E+00	2.02E+00	6.82E-01	1.69E+00	1.67E+00	1.70E-01	
9.30E+02	2.54E+00	2.00E+00	6.91E-01	1.69E+00	1.67E+00	3.40E-01	
9.60E+02	2.68E+00	2.16E+00	5.25E-01	1.69E+00	1.67E+00	3.71E-01	
9.90E+02	2.31E+00	1.73E+00	1.21E+00	1.86E+00	1.71E+00	2.40E-01	Karyofission
1.02E+03	2.30E+00	1.63E+00	1.37E+00	1.93E+00	1.79E+00	1.79E+00	

Cell 5

Time(s)	Mother Length	Daughter Length	Bridge Length	Mother Width	Daughter Width	Bridge Width	
0.00E+00	2.33E+00	1.50E-01	5.00E-02	2.15E+00	8.47E-01	8.47E-01	
3.00E+01	2.65E+00	1.50E-01	5.00E-02	2.15E+00	8.47E-01	8.47E-01	
6.00E+01	2.65E+00	1.50E-01	5.00E-02	2.32E+00	8.47E-01	8.47E-01	
9.00E+01	2.71E+00	1.50E-01	5.00E-02	2.17E+00	8.47E-01	8.47E-01	
1.20E+02	2.35E+00	5.99E-01	3.30E-01	2.17E+00	8.47E-01	8.30E-01	
1.50E+02	2.58E+00	5.99E-01	5.00E-02	2.30E+00	5.00E-01	3.30E-01	
1.80E+02	2.37E+00	4.90E-01	2.33E-01	2.05E+00	8.93E-01	6.60E-01	
2.10E+02	2.62E+00	5.00E-01	5.00E-02	1.94E+00	5.00E-01	3.40E-01	
2.40E+02	2.54E+00	6.05E-01	5.00E-02	1.99E+00	6.70E-01	5.00E-01	
2.70E+02	2.67E+00	7.07E-01	1.70E-01	1.99E+00	1.01E+00	8.30E-01	
3.00E+02	2.33E+00	6.82E-01	5.28E-01	2.00E+00	6.60E-01	6.60E-01	
3.30E+02	2.61E+00	5.99E-01	4.90E-01	1.90E+00	8.97E-01	7.38E-01	
3.60E+02	2.54E+00	6.89E-01	2.26E-01	2.17E+00	1.05E+00	1.01E+00	
3.90E+02	2.17E+00	8.93E-01	4.90E-01	2.17E+00	1.17E+00	8.30E-01	
4.20E+02	2.22E+00	8.30E-01	5.15E-01	1.86E+00	1.42E+00	8.47E-01	
4.50E+02	2.19E+00	8.30E-01	5.15E-01	2.06E+00	1.42E+00	8.30E-01	
4.80E+02	2.19E+00	8.47E-01	5.15E-01	2.00E+00	1.33E+00	1.18E+00	
5.10E+02	2.34E+00	1.00E+00	4.90E-01	2.17E+00	1.33E+00	1.01E+00	
5.40E+02	2.17E+00	1.34E+00	2.33E-01	2.22E+00	1.37E+00	8.30E-01	
5.70E+02	2.19E+00	1.34E+00	2.40E-01	2.03E+00	1.34E+00	8.47E-01	
6.00E+02	2.33E+00	1.21E+00	5.00E-01	2.16E+00	1.17E+00	4.67E-01	
6.30E+02	2.79E+00	1.21E+00	1.70E-01	1.85E+00	1.69E+00	1.16E+00	
6.60E+02	2.53E+00	1.50E+00	1.70E-01	1.73E+00	1.57E+00	1.00E+00	
6.90E+02	2.46E+00	1.42E+00	1.70E-01	1.94E+00	1.48E+00	8.45E-01	
7.20E+02	2.33E+00	1.49E+00	3.40E-01	2.00E+00	1.32E+00	8.30E-01	
7.50E+02	2.75E+00	1.50E+00	5.28E-01	1.79E+00	1.50E+00	8.45E-01	
7.80E+02	2.41E+00	1.53E+00	1.70E-01	1.99E+00	1.67E+00	8.47E-01	
8.10E+02	2.00E+00	2.16E+00	6.70E-01	2.19E+00	1.67E+00	3.30E-01	
8.40E+02	2.34E+00	2.00E+00	5.28E-01	1.99E+00	1.84E+00	1.70E-01	
8.70E+02	2.15E+00	1.67E+00	8.45E-01	2.00E+00	1.66E+00	1.60E-01	
9.00E+02	2.16E+00	1.83E+00	1.00E+00	1.89E+00	1.83E+00	1.60E-01	
9.30E+02	2.34E+00	1.84E+00	1.00E+00	1.85E+00	1.67E+00	1.60E-01	Karyofission
9.60E+02	2.33E+00	2.01E+00	1.17E+00	1.86E+00	1.69E+00	1.69E+00	
9.90E+02	2.33E+00	1.67E+00	1.34E+00	1.99E+00	1.50E+00	1.50E+00	
1.02E+03	2.19E+00	1.67E+00	1.67E+00	1.89E+00	1.66E+00	1.66E+00	

Cell 6

Time(s)	Mother Length	Daughter Length	Bridge Length	Mother Width	Daughter Width	Bridge Width	
0.00E+00	2.17E+00	1.50E-01	5.00E-02	2.16E+00	3.67E-01	3.67E-01	
3.00E+01	2.19E+00	1.50E-01	5.00E-02	2.17E+00	3.67E-01	3.67E-01	
6.00E+01	2.67E+00	1.50E-01	5.00E-02	2.02E+00	3.67E-01	3.67E-01	
9.00E+01	3.00E+00	1.50E-01	5.00E-02	1.86E+00	3.67E-01	3.67E-01	
1.20E+02	3.15E+00	1.50E-01	5.00E-02	2.19E+00	3.67E-01	3.67E-01	
1.50E+02	2.65E+00	8.47E-01	5.00E-02	1.99E+00	3.67E-01	3.67E-01	
1.80E+02	2.83E+00	6.70E-01	2.33E-01	1.83E+00	6.60E-01	3.30E-01	
2.10E+02	2.82E+00	8.93E-01	3.30E-01	1.99E+00	7.42E-01	3.67E-01	
2.40E+02	2.99E+00	8.93E-01	3.30E-01	2.00E+00	8.93E-01	3.67E-01	
2.70E+02	2.99E+00	1.01E+00	3.67E-01	2.00E+00	8.47E-01	5.99E-01	
3.00E+02	2.38E+00	1.66E+00	5.25E-01	2.10E+00	8.30E-01	3.30E-01	
3.30E+02	2.17E+00	2.15E+00	6.05E-01	2.05E+00	1.00E+00	3.40E-01	
3.60E+02	2.49E+00	1.50E+00	6.82E-01	1.99E+00	1.16E+00	5.28E-01	
3.90E+02	2.49E+00	1.50E+00	6.05E-01	1.95E+00	1.06E+00	3.76E-01	
4.20E+02	2.73E+00	1.67E+00	3.30E-01	1.73E+00	1.21E+00	3.80E-01	
4.50E+02	2.65E+00	1.67E+00	3.30E-01	1.66E+00	1.34E+00	5.00E-01	
4.80E+02	2.49E+00	1.84E+00	6.82E-01	1.83E+00	1.32E+00	2.33E-01	
5.10E+02	2.49E+00	2.00E+00	5.00E-01	1.84E+00	1.16E+00	3.30E-01	
5.40E+02	2.34E+00	1.99E+00	6.60E-01	1.99E+00	1.50E+00	5.00E-01	Karyofission
5.70E+02	2.33E+00	1.66E+00	1.16E+00	1.83E+00	1.51E+00	1.51E+00	
6.00E+02	2.17E+00	1.67E+00	1.34E+00	1.82E+00	1.66E+00	1.66E+00	
6.30E+02	2.16E+00	1.84E+00	1.33E+00	1.84E+00	1.49E+00	1.49E+00	
6.60E+02	2.00E+00	1.67E+00	1.84E+00	1.67E+00	1.84E+00	1.84E+00	

Cell 7

Time(s)	Mother Length	Daughter Length	Bridge Length	Mother Width	Daughter Width	Bridge Width
0.00E+00	2.16E+00	1.50E-01	5.00E-02	2.51E+00	5.28E-01	5.28E-01
3.00E+01	2.34E+00	1.50E-01	5.00E-02	2.35E+00	5.28E-01	5.28E-01
6.00E+01	2.90E+00	1.50E-01	5.00E-02	2.05E+00	5.28E-01	5.28E-01
9.00E+01	2.68E+00	1.50E-01	5.00E-02	2.22E+00	5.28E-01	5.28E-01
1.20E+02	3.15E+00	1.50E-01	5.00E-02	1.95E+00	5.28E-01	5.28E-01
1.50E+02	2.99E+00	3.40E-01	5.00E-02	2.08E+00	5.28E-01	5.28E-01
1.80E+02	2.72E+00	9.69E-01	5.00E-02	2.03E+00	8.28E-01	8.28E-01
2.10E+02	2.75E+00	5.99E-01	5.00E-02	2.08E+00	5.25E-01	5.25E-01
2.40E+02	3.14E+00	6.05E-01	5.00E-02	2.03E+00	6.05E-01	6.05E-01
2.70E+02	2.77E+00	7.47E-01	2.33E-01	2.17E+00	8.36E-01	4.67E-01
3.00E+02	2.96E+00	7.07E-01	2.26E-01	2.13E+00	7.07E-01	5.28E-01
3.30E+02	2.46E+00	8.28E-01	3.67E-01	1.94E+00	9.64E-01	5.99E-01
3.60E+02	3.15E+00	6.60E-01	5.25E-01	1.84E+00	1.34E+00	8.45E-01
3.90E+02	3.18E+00	1.17E+00	2.26E-01	1.99E+00	1.34E+00	5.00E-01
4.20E+02	2.83E+00	1.34E+00	2.33E-01	2.00E+00	1.17E+00	6.70E-01
4.50E+02	2.18E+00	1.50E+00	3.80E-01	2.16E+00	1.33E+00	5.28E-01
4.80E+02	2.38E+00	1.17E+00	5.99E-01	2.17E+00	1.54E+00	7.51E-01
5.10E+02	2.55E+00	1.69E+00	3.71E-01	1.83E+00	1.53E+00	5.25E-01
5.40E+02	2.51E+00	2.17E+00	2.40E-01	1.67E+00	1.54E+00	3.67E-01
5.70E+02	2.16E+00	2.00E+00	6.70E-01	2.11E+00	1.68E+00	3.30E-01
6.00E+02	2.33E+00	2.03E+00	8.47E-01	2.00E+00	1.84E+00	2.33E-01
6.30E+02	2.63E+00	1.85E+00	8.45E-01	1.58E+00	1.67E+00	2.26E-01
6.60E+02	2.74E+00	1.73E+00	7.38E-01	1.48E+00	1.73E+00	3.76E-01
6.90E+02	2.91E+00	2.05E+00	8.30E-01	1.37E+00	1.90E+00	3.30E-01
7.20E+02	2.59E+00	2.16E+00	1.16E+00	1.64E+00	1.64E+00	1.70E-01
7.50E+02	2.16E+00	2.10E+00	1.37E+00	1.79E+00	1.79E+00	2.40E-01
7.80E+02	2.26E+00	2.26E+00	1.00E+00	1.63E+00	1.69E+00	5.28E-01
8.10E+02	2.63E+00	2.05E+00	1.21E+00	1.64E+00	1.73E+00	1.60E-01
8.40E+02	2.51E+00	2.19E+00	1.05E+00	1.69E+00	1.69E+00	2.33E-01
8.70E+02	2.38E+00	2.19E+00	1.00E+00	1.69E+00	1.54E+00	2.33E-01
9.00E+02	2.26E+00	2.22E+00	1.04E+00	1.90E+00	1.51E+00	1.60E-01
9.30E+02	2.38E+00	2.18E+00	1.37E+00	1.57E+00	1.67E+00	1.60E-01
9.60E+02	2.62E+00	2.21E+00	1.22E+00	1.63E+00	1.69E+00	5.99E-01
9.90E+02	2.62E+00	2.22E+00	1.50E+00	1.56E+00	1.69E+00	5.00E-01
1.02E+03	2.58E+00	2.41E+00	1.27E+00	1.57E+00	1.37E+00	3.30E-01
1.05E+03	2.37E+00	2.25E+00	1.58E+00	1.53E+00	1.79E+00	1.70E-01
1.08E+03	2.37E+00	2.41E+00	1.58E+00	1.79E+00	1.73E+00	3.71E-01
1.11E+03	2.26E+00	1.89E+00	2.05E+00	1.63E+00	1.73E+00	3.40E-01
1.14E+03	2.31E+00	2.05E+00	2.22E+00	1.73E+00	1.69E+00	1.70E-01
1.17E+03	2.31E+00	1.73E+00	1.90E+00	1.58E+00	1.73E+00	1.70E-01
1.20E+03	2.31E+00	1.88E+00	1.54E+00	1.64E+00	1.73E+00	1.70E-01
Time(s)	2.16E+00	1.88E+00	1.18E+00	1.63E+00	1.73E+00	3.30E-01

1.00E+00	2.23E+00	1.86E+00	1.17E+00	1.64E+00	1.86E+00	3.31E-01	
3.10E+01	2.31E+00	1.73E+00	1.34E+00	1.79E+00	1.69E+00	1.60E-01	
6.10E+01	2.05E+00	1.86E+00	1.50E+00	1.79E+00	1.86E+00	1.60E-01	
9.10E+01	2.21E+00	1.86E+00	1.50E+00	1.95E+00	1.73E+00	1.60E-01	Karyofission
1.21E+02	2.06E+00	1.79E+00	1.73E+00	1.95E+00	2.05E+00	2.05E+00	
1.51E+02	2.21E+00	1.73E+00	1.69E+00	1.89E+00	1.89E+00	1.89E+00	
1.81E+02	2.01E+00	1.73E+00	2.00E+00	1.88E+00	1.69E+00	1.69E+00	

Cell 8

Time(s)	Mother Length	Daughter Length	Bridge Length	Mother Width	Daughter Width	Bridge Width	
0.00E+00	2.59E+00	1.50E-01	5.00E-02	2.17E+00	7.38E-01	7.38E-01	
3.00E+01	2.83E+00	1.50E-01	5.00E-02	1.94E+00	7.38E-01	7.38E-01	
6.00E+01	2.63E+00	7.42E-01	3.76E-01	1.94E+00	7.38E-01	3.71E-01	
9.00E+01	2.85E+00	9.69E-01	2.33E-01	2.03E+00	5.25E-01	2.33E-01	
1.20E+02	2.13E+00	1.41E+00	1.17E+00	2.03E+00	5.99E-01	4.81E-01	
1.50E+02	2.24E+00	1.34E+00	9.69E-01	2.00E+00	9.40E-01	3.76E-01	
1.80E+02	2.99E+00	1.11E+00	2.33E-01	1.90E+00	1.11E+00	6.05E-01	
2.10E+02	2.70E+00	1.27E+00	3.76E-01	1.94E+00	1.06E+00	6.05E-01	
2.40E+02	2.70E+00	1.34E+00	2.33E-01	2.00E+00	1.06E+00	8.30E-01	
2.70E+02	2.47E+00	2.01E+00	2.33E-01	1.94E+00	1.21E+00	5.28E-01	
3.00E+02	2.82E+00	1.79E+00	4.74E-01	1.63E+00	1.42E+00	5.28E-01	
3.30E+02	2.31E+00	1.94E+00	1.70E-01	2.01E+00	1.37E+00	5.99E-01	
3.60E+02	2.39E+00	1.79E+00	3.71E-01	1.94E+00	1.42E+00	7.47E-01	
3.90E+02	2.49E+00	1.86E+00	3.80E-01	1.94E+00	1.58E+00	7.38E-01	
4.20E+02	2.26E+00	1.94E+00	3.71E-01	1.89E+00	1.57E+00	7.47E-01	
4.50E+02	2.03E+00	2.07E+00	4.81E-01	1.71E+00	1.57E+00	5.28E-01	
4.80E+02	2.37E+00	1.90E+00	5.99E-01	1.88E+00	1.66E+00	4.74E-01	
5.10E+02	2.22E+00	2.16E+00	6.05E-01	2.01E+00	1.58E+00	5.99E-01	
5.40E+02	2.31E+00	2.07E+00	6.05E-01	2.01E+00	1.71E+00	5.99E-01	
5.70E+02	2.38E+00	1.79E+00	7.51E-01	1.80E+00	1.69E+00	2.40E-01	
6.00E+02	2.38E+00	2.01E+00	7.42E-01	2.01E+00	1.57E+00	2.40E-01	
6.30E+02	2.25E+00	2.10E+00	6.91E-01	1.69E+00	1.69E+00	3.40E-01	
6.60E+02	2.38E+00	2.21E+00	1.00E+00	1.73E+00	1.67E+00	1.70E-01	
6.90E+02	2.32E+00	2.10E+00	1.01E+00	1.69E+00	1.67E+00	1.60E-01	Karyofission
7.20E+02	2.11E+00	1.86E+00	1.57E+00	1.86E+00	1.79E+00	1.79E+00	
7.50E+02	2.10E+00	1.84E+00	1.90E+00	1.86E+00	1.57E+00	1.57E+00	
7.80E+02	2.26E+00	2.05E+00	1.67E+00	1.95E+00	1.85E+00	1.85E+00	

Cell 9

Time(s)	Mother Length	Daughter Length	Bridge Length	Mother Width	Daughter Width	Bridge Width
0.00E+00	2.64E+00	1.50E-01	5.00E-02	2.19E+00	8.47E-01	8.47E-01
3.00E+01	2.30E+00	1.50E-01	5.00E-02	2.55E+00	8.47E-01	8.47E-01
6.00E+01	2.58E+00	1.50E-01	5.00E-02	2.16E+00	8.47E-01	8.47E-01
9.00E+01	2.83E+00	1.50E-01	5.00E-02	2.32E+00	8.47E-01	8.47E-01
1.20E+02	3.20E+00	1.50E-01	5.00E-02	2.00E+00	8.47E-01	8.47E-01
1.50E+02	3.55E+00	1.50E-01	5.00E-02	2.02E+00	8.47E-01	8.47E-01
1.80E+02	3.99E+00	1.50E-01	5.00E-02	2.03E+00	8.47E-01	8.47E-01
2.10E+02	4.18E+00	1.50E-01	5.00E-02	1.84E+00	8.47E-01	8.47E-01
2.40E+02	4.20E+00	1.50E-01	5.00E-02	1.84E+00	8.47E-01	8.47E-01
2.70E+02	4.16E+00	1.50E-01	5.00E-02	1.86E+00	8.47E-01	8.47E-01
3.00E+02	4.16E+00	1.50E-01	5.00E-02	1.67E+00	8.47E-01	8.47E-01
3.30E+02	4.49E+00	1.50E-01	5.00E-02	1.89E+00	8.47E-01	8.47E-01
3.60E+02	3.65E+00	6.68E-01	5.00E-02	1.84E+00	8.47E-01	8.47E-01
3.90E+02	3.32E+00	1.17E+00	3.30E-01	1.66E+00	9.90E-01	5.00E-01
4.20E+02	2.38E+00	1.66E+00	6.82E-01	1.79E+00	9.64E-01	7.38E-01
4.50E+02	3.10E+00	1.76E+00	5.00E-02	1.73E+00	9.69E-01	6.79E-01
4.80E+02	2.26E+00	2.10E+00	3.71E-01	1.69E+00	1.34E+00	5.00E-01
5.10E+02	2.75E+00	1.86E+00	3.30E-01	1.79E+00	1.33E+00	8.47E-01
5.40E+02	2.94E+00	2.08E+00	1.60E-01	1.93E+00	1.48E+00	6.82E-01
5.70E+02	2.79E+00	2.00E+00	5.00E-02	1.86E+00	1.42E+00	5.00E-01
6.00E+02	2.99E+00	2.10E+00	1.70E-01	1.67E+00	1.53E+00	5.00E-01
6.30E+02	2.79E+00	1.99E+00	3.67E-01	1.73E+00	1.67E+00	5.00E-01
6.60E+02	1.73E+00	2.01E+00	1.34E+00	1.69E+00	1.48E+00	5.19E-01
6.90E+02	3.27E+00	2.00E+00	3.71E-01	1.67E+00	1.67E+00	3.30E-01
7.20E+02	2.94E+00	2.31E+00	5.25E-01	1.69E+00	1.57E+00	4.90E-01
7.50E+02	2.33E+00	2.02E+00	1.01E+00	2.00E+00	1.50E+00	3.30E-01
7.80E+02	2.66E+00	2.17E+00	7.38E-01	1.83E+00	1.50E+00	6.70E-01
8.10E+02	2.51E+00	2.17E+00	1.01E+00	1.85E+00	1.67E+00	1.70E-01
8.40E+02	2.21E+00	2.08E+00	1.51E+00	1.95E+00	1.50E+00	1.60E-01
8.70E+02	2.58E+00	2.31E+00	1.12E+00	1.79E+00	1.58E+00	3.40E-01
9.00E+02	2.42E+00	2.62E+00	1.04E+00	1.73E+00	1.67E+00	2.33E-01
9.30E+02	2.37E+00	2.02E+00	1.90E+00	1.85E+00	1.84E+00	1.60E-01
9.60E+02	2.50E+00	2.05E+00	1.79E+00	1.83E+00	1.67E+00	3.30E-01
9.90E+02	2.41E+00	1.89E+00	1.90E+00	1.95E+00	1.79E+00	4.90E-01
1.02E+03	2.47E+00	1.79E+00	2.05E+00	1.89E+00	1.73E+00	3.71E-01
1.05E+03	2.42E+00	1.85E+00	2.22E+00	1.73E+00	1.79E+00	3.30E-01
1.08E+03	2.01E+00	2.10E+00	2.58E+00	1.73E+00	1.73E+00	3.71E-01
1.11E+03	2.08E+00	1.85E+00	2.91E+00	1.79E+00	1.73E+00	3.71E-01
1.14E+03	2.30E+00	2.05E+00	2.63E+00	1.79E+00	1.73E+00	2.33E-01
1.17E+03	2.10E+00	2.02E+00	2.68E+00	1.79E+00	1.73E+00	3.30E-01
1.20E+03	1.94E+00	2.04E+00	2.68E+00	1.95E+00	1.90E+00	5.00E-01
Time(s)	2.26E+00	2.00E+00	2.22E+00	1.94E+00	1.89E+00	2.40E-01

1.00E+00	2.17E+00	1.71E+00	2.10E+00	2.00E+00	1.73E+00	1.60E-01	
3.10E+01	2.17E+00	2.01E+00	1.53E+00	1.95E+00	1.69E+00	1.60E-01	
6.10E+01	1.95E+00	1.95E+00	1.42E+00	1.95E+00	1.78E+00	1.84E-01	Karyofission
9.10E+01	1.89E+00	2.01E+00	1.42E+00	2.06E+00	1.71E+00	1.71E+00	
1.21E+02	2.01E+00	1.95E+00	1.63E+00	1.95E+00	1.89E+00	1.89E+00	
1.51E+02	2.01E+00	1.79E+00	1.42E+00	2.16E+00	1.63E+00	1.63E+00	
1.81E+02	1.79E+00	1.79E+00	1.64E+00	1.79E+00	1.90E+00	1.90E+00	
2.11E+02	2.00E+00	2.10E+00	1.37E+00	1.89E+00	1.86E+00	1.86E+00	

Cell 10

Time(s)	Mother Length	Daughter Length	Bridge Length	Mother Width	Daughter Width	Bridge Width	
0.00E+00	2.72E+00	1.50E-01	5.00E-02	2.36E+00	5.99E-01	5.99E-01	
3.00E+01	2.82E+00	1.50E-01	5.00E-02	2.03E+00	5.99E-01	5.99E-01	
6.00E+01	2.86E+00	1.50E-01	5.00E-02	2.03E+00	5.99E-01	5.99E-01	
9.00E+01	3.41E+00	1.50E-01	5.00E-02	2.11E+00	5.99E-01	5.99E-01	
1.20E+02	3.88E+00	1.50E-01	5.00E-02	2.00E+00	5.99E-01	5.99E-01	
1.50E+02	3.92E+00	1.50E-01	5.00E-02	2.03E+00	5.99E-01	5.99E-01	
1.80E+02	3.47E+00	7.00E-01	5.00E-02	2.03E+00	5.99E-01	5.99E-01	
2.10E+02	3.45E+00	7.07E-01	5.00E-02	1.88E+00	7.07E-01	7.07E-01	
2.40E+02	3.89E+00	2.26E-01	5.00E-02	1.89E+00	7.47E-01	7.47E-01	
2.70E+02	3.77E+00	4.74E-01	5.00E-02	1.87E+00	5.99E-01	5.99E-01	
3.00E+02	4.00E+00	3.67E-01	5.00E-02	2.11E+00	7.38E-01	7.38E-01	
3.30E+02	4.00E+00	4.74E-01	5.00E-02	2.00E+00	7.51E-01	7.51E-01	
3.60E+02	3.32E+00	9.69E-01	1.70E-01	1.79E+00	1.06E+00	5.19E-01	
3.90E+02	3.43E+00	1.41E+00	1.60E-01	1.80E+00	9.40E-01	9.40E-01	
4.20E+02	2.94E+00	1.53E+00	1.70E-01	1.89E+00	1.06E+00	9.48E-01	
4.50E+02	2.81E+00	1.34E+00	4.74E-01	1.76E+00	1.30E+00	7.51E-01	
4.80E+02	2.81E+00	1.57E+00	3.71E-01	1.88E+00	1.53E+00	8.28E-01	
5.10E+02	2.82E+00	1.65E+00	3.67E-01	1.88E+00	1.41E+00	8.28E-01	
5.40E+02	2.66E+00	1.66E+00	4.94E-01	1.76E+00	1.66E+00	8.22E-01	
5.70E+02	2.35E+00	2.03E+00	3.71E-01	2.03E+00	1.57E+00	7.47E-01	
6.00E+02	2.17E+00	2.35E+00	6.60E-01	2.09E+00	1.67E+00	5.28E-01	
6.30E+02	2.39E+00	1.80E+00	5.99E-01	1.77E+00	1.79E+00	8.28E-01	
6.60E+02	2.39E+00	2.00E+00	4.74E-01	1.76E+00	1.77E+00	7.00E-01	
6.90E+02	2.62E+00	1.86E+00	4.74E-01	1.76E+00	1.57E+00	3.71E-01	
7.20E+02	2.72E+00	2.11E+00	5.25E-01	1.76E+00	1.66E+00	3.67E-01	
7.50E+02	2.76E+00	2.17E+00	4.67E-01	1.89E+00	1.65E+00	3.80E-01	
7.80E+02	2.62E+00	2.16E+00	6.05E-01	1.77E+00	1.66E+00	2.33E-01	
8.10E+02	2.63E+00	2.23E+00	5.99E-01	2.11E+00	1.76E+00	2.33E-01	
8.40E+02	2.49E+00	2.12E+00	9.69E-01	1.49E+00	1.89E+00	3.67E-01	
8.70E+02	2.36E+00	2.03E+00	1.19E+00	1.88E+00	1.66E+00	3.67E-01	
9.00E+02	2.49E+00	2.00E+00	1.12E+00	1.87E+00	1.80E+00	2.33E-01	
9.30E+02	2.47E+00	2.12E+00	1.41E+00	1.53E+00	1.80E+00	3.76E-01	
9.60E+02	2.35E+00	2.13E+00	1.48E+00	1.88E+00	1.86E+00	4.67E-01	
9.90E+02	2.47E+00	2.00E+00	1.73E+00	2.00E+00	1.71E+00	3.80E-01	
1.02E+03	2.24E+00	2.11E+00	2.00E+00	1.88E+00	1.71E+00	2.40E-01	
1.05E+03	2.36E+00	2.13E+00	1.86E+00	1.77E+00	1.57E+00	3.80E-01	
1.08E+03	2.13E+00	1.86E+00	2.37E+00	1.88E+00	1.80E+00	4.67E-01	
1.11E+03	2.23E+00	2.25E+00	2.17E+00	1.88E+00	1.79E+00	2.40E-01	Karyofission
1.14E+03	2.11E+00	1.90E+00	2.44E+00	1.88E+00	1.77E+00	1.77E+00	
1.17E+03	2.26E+00	2.03E+00	2.44E+00	2.13E+00	1.80E+00	1.80E+00	
1.20E+03	2.26E+00	2.00E+00	2.37E+00	2.00E+00	1.90E+00	1.90E+00	
Time(s)	2.13E+00	2.00E+00	1.63E+00	2.00E+00	2.00E+00	2.00E+00	

1.00E+00	2.00E+00	1.76E+00	1.64E+00	1.88E+00	1.90E+00	1.90E+00
3.10E+01	2.24E+00	2.67E+00	1.23E+00	2.00E+00	1.79E+00	1.79E+00
6.10E+01	2.35E+00	2.23E+00	1.21E+00	1.89E+00	1.79E+00	1.79E+00
9.10E+01	2.11E+00	2.00E+00	1.48E+00	1.99E+00	1.66E+00	1.66E+00
