# Supplementary material for: Asymmetrical Inheritance of Plasmids Depends on Dynamic Cellular Geometry and Volume Exclusion Effects
Source: PLoS One. 2015 Oct 15;10(10):e0139443. doi: 10.1371/journal.pone.0139443 (PMC4607505; doi:10.1371/journal.pone.0139443)
Supplement: S1 Text — (PDF) [file pone.0139443.s006.pdf]

S1 Text - Denton J. A., Ghosh, A. and Marquez-Lago, T.T.

Individual diffusion constants estimated per cell, chosen according to their stage in the cell cycle. Coordinates of the plasmid were tracked every 5 seconds in ImageJ. For each cell, a distribution of 5-second jump sizes was calculated in a 2D plane. Assuming Brownian motion, we have that the diffusion coefficient, *D*, is then given by: $D = \frac{\langle x^2 \rangle}{2d\Delta t}$, where $\langle x^2 \rangle$ is the average jump size, Δt is the time interval (5 seconds) and *d* is the dimensionality (2, since we only track the movement in a plane). Particle jumps near nuclear membranes were ignored, since the input for simulations should be a free diffusion coefficient. This leads to the following diffusion coefficients, all in $\mu m^2 / s$.

**Interphase**	**Anaphase**
0.0016	0.0035
0.0023	0.0036
0.0035	0.0020
0.0022	0.0010
0.0043	0.0032
0.0014	0.0040
0.0026	0.0027
0.0029	0.0034
0.0028	0.0025
0.0021	0.0026
0.0022	0.0031
0.0027	0.0022
0.0022	0.0024
0.0020	0.0043
0.0016	0.0022
0.0016	0.0045
0.0026	0.0046
0.0022	0.0026
0.0017	0.0037
0.0030	0.0037
0.0029	0.0023
0.0043	0.0024
0.0031	0.0037
0.0028	0.0031

The average of all such cells is 0.0027 $\mu m^2 / s$. The average of interphase cells is 0.0025 $\mu m^2 / s$; the average of anaphase cells is 0.0031 $\mu m^2 / s$.
