# Supplementary material for: Asymmetrical Inheritance of Plasmids Depends on Dynamic Cellular Geometry and Volume Exclusion Effects
Source: PLoS One. 2015 Oct 15;10(10):e0139443. doi: 10.1371/journal.pone.0139443 (PMC4607505; doi:10.1371/journal.pone.0139443)
Supplement: S2 Text — (PDF) [file pone.0139443.s007.pdf]

S2 Text - Denton J. A., Ghosh, A. and Marquez-Lago, T.T.

Simulation methodology

The methodology is described in detail in Reference [28] of the manuscript, also included below. We provide a brief overview here, and refer readers there for a full discussion with validation of the technique.

A general yeast nuclear geometry was represented by two oblate spheroids connected by a cylindrical bridge. Each oblate spheroid was truncated in such a way such that the cylindrical bridge would adjoin it without creating any gaps.

Thus, we assume a rotationally symmetric domain for the nuclear geometry. The size of each oblate spheroid could thus be described by two numbers describing the major and minor axes. The cylinder adjoining them could be described by the cylindrical length and diameter. The figure below illustrates a sample geometry projected onto a 2D plane.

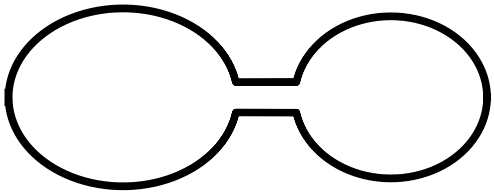

The parameters describing the geometry were measured by confocal microscopy at 30 second intervals. In order to interpolate between the geometries smoothly, the parameters were linearly interpolated between the 30 second time steps. The geometries were constructed in such a way so that the centre of mass of the geometry was at the origin at every time step.

At every time step, a particle was propagated according to a Gaussian kernel. In case the particle would overlap with the boundary, a vector connecting the particle position to the closest point on the boundary was constructed. The particle was then moved in the direction of this vector such that the particle would be completely contained within the boundary once more.

The midpoint of the bridge was taken to be the demarcation zone between the mother and the daughter.

[28] Ghosh A, Marquez-Lago TT (2015) Simulating Stochastic Reaction-Diffusion Systems on and within Moving Boundaries. PLoS ONE 10: e0133401. doi:10.1371/journal.pone.0133401.
